# Characterising acute ischaemic stroke thrombi: insights from histology, imaging and emerging impedance-based technologies

**DOI:** 10.1136/svn-2021-001038

**Published:** 2022-03-03

**Authors:** Smita Patil, Jean Darcourt, Pierluca Messina, Franz Bozsak, Christophe Cognard, Karen Doyle

**Affiliations:** 1 CÚRAM, SFI Research Centre for Medical Devices, National University of Ireland Galway, Galway, Ireland; 2 Neuroradiology, CHU de Toulouse, Toulouse, France; 3 Sensome, Massy, France; 4 Physiology, National University of Ireland Galway, Galway, Ireland

**Keywords:** stroke, thrombectomy, intervention, thrombolysis

## Abstract

Treatment of acute ischaemic stroke (AIS) focuses on rapid recanalisation of the occluded artery. In recent years, advent of mechanical thrombectomy devices and new procedures have accelerated the analysis of thrombi retrieved during the endovascular thrombectomy procedure. Despite ongoing developments and progress in AIS imaging techniques, it is not yet possible to conclude definitively regarding thrombus characteristics that could advise on the probable efficacy of thrombolysis or thrombectomy in advance of treatment. Intraprocedural devices with dignostic capabilities or new clinical imaging approaches are needed for better treatment of AIS patients. In this review, what is known about the composition of the thrombi that cause strokes and the evidence that thrombus composition has an impact on success of acute stroke treatment has been examined. This review also discusses the evidence that AIS thrombus composition varies with aetiology, questioning if suspected aetiology could be a useful indicator to stroke physicians to help decide the best acute course of treatment. Furthermore, this review discusses the evidence that current widely used radiological imaging tools can predict thrombus composition. Further use of new emerging technologies based on bioimpedance, as imaging modalities for diagnosing AIS and new medical device tools for detecting thrombus composition in situ has been introduced. Whether bioimpedance would be beneficial for gaining new insights into in situ thrombus composition that could guide choice of optimum treatment approach is also reviewed.

## Introduction

Occlusion of a cerebral artery by a thrombus results in acute ischaemic stroke (AIS). Treatment of AIS aims to recanalise the occluded artery, promptly and efficiently, either by intravenous thrombolysis via recombinant tissue plasminogen activator (r-tPA) or mechanical removal of the thrombus via endovascular thrombectomy (EVT). In most countries, less than 15% of AIS patients are able to avail r-tPA treatment^1 2^ as it must be administered within 4.5 hours of stroke onset to minimise risk of cerebral haemorrhage. Of those who are treated with r-tPA, successful reperfusion is achieved in less than half of cases.^3^ The reperfusion failure has been attributed to factors such as excess thrombus burden or inadequate dose of thrombolytic drug; aged thrombus; thrombus location; thrombolytic drug resistance. Mechanical thrombectomy is becoming more mainstream, although it is only available in stroke centres with trained neurointerventionalists. With mechanical thrombectomy, successful recanalisation is attained in 70%–80% of cases.^4^ The reasons behind the failure of recanalisation in some patients are not fully understood. Other than vascular access, composition of thrombus is likely to be an important factor.^5^ Although, thrombus composition and characteristics are not, at present considered to any great extent in treatment decision making, better understanding of the thrombus prior to intervention could help in achieving successful recanalisation and reduce adverse secondary events via selection of appropriate thrombolytic and/or endovascular strategy for intervention.

### AIS thrombus composition

Until recently, there was limited availability of the thrombi that cause strokes. Available samples were mainly limited to occasional postmortem tissue and the clots removed in the course of thrombectomy device clinical trials. Since the success of clinical trials demonstrating effectiveness of mechanical thrombectomy in AIS patients with large vessel occlusions, more occluding thrombi removed during EVT procedures are available for analysis, allowing us to gain insights into thrombus composition.

Perhaps the most characteristic feature of AIS thrombi retrieved by thrombectomy is the marked heterogeneity observed. A range of studies have described gross characteristics such as size, shape, morphology, consistency (soft, solid, elastic).^6–10^ However, there is no consistency in the way thrombi are described in these studies. Thrombi can be classified based on main components: red blood cells (RBCs), white blood cells (WBCs), fibrin and platelets.^11^ Some studies do not report the exact composition of thrombi, instead fibrin rich thrombi are called white or hard, RBC rich thrombi as red, erythrocytic or soft, and calcified thrombi as aged.^7 12 13^ Sometimes, deposition patterns of RBCs and fibrin, in various regions of thrombus are also used for thrombus description. Thrombi with fibrin and RBCs deposited in layers are described as layered thrombi and if deposited in distinctive winding pattern, thrombi are called serpentine.^7 12^ A recent review has summarised all the studies with thrombus categorisation based on dominant components.^14^

Using H&E staining, AIS thrombi can be broadly classified into three subtypes, RBC rich, fibrin rich, or mixed. H&E staining cannot differentiate between fibrin and platelets. Nonetheless, using H&E staining, initial studies found that the composition of AIS thrombi is highly variable (table 1).^6–10^

**Table 1:** Thrombus composition and aetiology

Ref. (year)	No of patients	% of patients w.r.t aetiology	Staining technique	Main findings
Marder *et al* 7 2006	25	LAA:16 CE:64 ODE:12 UDE:8	H&E	Similar histological components in CE and LAA
Ogata *et al* 20 2008	142	LAA:12 CE:75 SVO:1 ODE:9 UDE:3	H&E, Masson’s trichrome, Mallory’s phosphotungstic acid haematoxylin	Most LAA thrombi are fibrin and platelet rich at plaque site and become fibrin and RBC rich while occluding brain arteries CE strokes have RBC rich thrombi
Niesten *et al* 19 2014	22	LAA:36 CE:27 ODE:14 UDE:23	H&E, Mallory’s phosphotungstic acid- haematoxylin (fibrin) IHC: glycophorin A (RBCs) and CD31 (platelets)	LAA thrombi are RBC rich No differences in platelets and fibrin content between stroke subtypes
Boeckh-Behrens *et al* 9 2014	34	LAA:9 CE:47 ODE:18 UDE:26	H&E, Elastica van Gieson staining	Higher percentage of WBCs in the thrombus indicates organised thrombi of CE origin
Kim *et al* 35 2015	37	LAA:22 CE:59 UDE:19	H&E, IHC: CD61(platelet glycoprotein IIIa)	CE thrombi are RBC rich and have lower fibrin content compared with LAA thrombi
Simons *et al* 23 2015	40	CE: 53	H&E IHC:CD34	Comparatively higher RBC content in CE thrombi No significant association between CE stroke and thrombus composition
Ahn *et al* 15 2016	36	LAA: 22 CE: 61 UDE: 17	H&E, MSB IHC: CD42b (platelet glycoprotein Ib)	LAA thrombi were RBC rich and CE thrombi were fibrin rich with platelets clustered within the rich fibrin UDE thrombi (5/6) had histologic features and composition like CE thrombi
Boeckh-Behrens *et al* 8 2016	137	LAA:16 CE:49 ODE:8 UDE:27	H&E	CE thrombi had higher proportions of fibrin/platelets, less erythrocytes, and more leucocytes than noncardioembolic thrombi UDE strokes had thrombus histology and interventional and clinical outcome parameters similar to CE strokes
Dargazanli *et al* 36 2016	54	LAA:19 CE:46 ODE:24 UDE:11	H&E, IHC: CD3 (T cells)	High CD3 +T cells count associated with LAA thrombi
Schuhmann *et al* 12 2016	37	LAA: 37 CE:51 UDE:12	H&E, MSB IHC: CD4 (T cells), CD68 (monocytes) and vWF	Stroke subtype and clinical outcome had no association with immune cell or platelet % or distribution in thrombi RBC rich clots had higher T cells and monocytes
Sporns *et al* 32 2017	187	LAA:19 CE:41 ODE:6 UDE:34	H&E, Elastica van Gieson, Prussian blue IHC: CD3, CD20, and CD68/KiM1P	CE thrombi are fibrin/platelet rich with more WBCs than non CE thrombi (LAA+ODE) Cryptogenic strokes and CE strokes showed similar thrombus histology The IHC parameters showed no noticeable differences between stroke subtypes
Maekawa *et al* 33 2018	43	LAA: 12 CE: 70 ODE: 2 UDE: 16	H&E	RBC rich thrombi are associated with non CE aetiology
Berndt *et al* 57 2018	133	LAA: 12 CE: 53 ODE: 2 UDE: 33	H&E (NCCT and CTA for clot perviousness)	Pervious thrombi are fibrin/platelet rich. Pervious thrombi were significantly associated with CE compared with non-CE thrombi
Shin *et al* 31 2018	37	LAA:19 CE:59 UDE:22	H&E	RBC-rich clots associated with CE stroke aetiology Fibrin/platelet rich thrombi were more frequent in LAA and undetermined subtypes whereas mixed type was more frequent in CE patients
Fitzgerald *et al* 34 2019	105	LAA:19 CE:50 ODE:11 UDE:20	H&E, MSB	Platelet-rich clots and % of platelet content significantly higher in LAA than CE thrombi No correlation between stroke aetiology and the other major thrombus components
Staessens *et al* 5 2020	177 thrombi	NA	H&E, MSB, Feulgen’s reaction (DNA staining) IHC and IFC: vWF, platelets (GPIbα), fibrin, leukocytes (CD45), RBCs (autofluorescence)	AIS thrombi composed of two main types of areas: RBC rich areas and platelet rich areas. RBC-rich areas are with densely packed RBCs within a meshwork of thin fibrin strands, and very few nucleated cells or vWF. Dense fibrin structures together with vWF, delineate platelet-rich areas. Leukocytes and DNA chiefly present at the interface between RBC rich and platelet rich areas

*All studies used TOAST classification of stroke except for reference 20.

CE, cardioembolism; CTA, CT angiography; IA, intra-arterial; IFC, immunofluorescence staining; IHC, immunohistochemical staining; LAA, large-artery atherosclerosis; MSB, Martius scarlet blue; NCCT, non-contrast CT; ODE, stroke of other determined aetiology; RBC, red blood cell; SVO, small-vessel occlusion; SVS, susceptibility vessel sign; TOAST, Trial of Org 10 172 in Acute Stroke Treatment; UDE, stroke of undetermined aetiology; vWF, von Willebrand factor; WBC, white blood cell.

Some studies have employed Martius scarlet blue (MSB) staining, which provides better differentiation between fibrin and platelet components.^11 12 15^ Using immunohistochemical staining against CD42b (platelet glycoprotein Ib), Fitzgerald *et al* demonstrated that MSB staining can reliably identify platelet-rich areas.^11^ A recent study has demonstrated that platelet-rich thrombi are also von Willebrand factor (vWF)-rich.^16^ Other histological stains have also been used for identifying specific components, for example, Masson’s Trichrome (collagen),^10 17^ von Kossa (calcification),^18^ Elastica van-Gieson (elastic fibres and collagen),^9 17^ Mallory’s phosphotungstic acid haematoxylin (fibrin and collagen)^19 20^ and have provided additional insights into the thrombus composition.

CD42b immunohistochemical staining was used to study the platelet organisation within thrombi. Platelets were observed covering the fibrin layers, located at the periphery of RBC-rich arteriogenic thrombi or were clustered within fibrin rich cardioembolic thrombi.^15^ Histological and immunofluorescent analysis identified two main types of areas within thrombi, RBC-rich areas and platelet-rich areas.^5^ Dense fibrin structures were shown to delineate platelet-rich areas within thrombi. Leucocytes and DNA were chiefly found at the interface between RBC-rich and platelet-rich areas. RBC-rich areas had densely packed RBCs within a meshwork of thin fibrin strands, and very few nucleated cells or vWF.^5^

Activated neutrophils release histones and granule proteins embedded in web-like assembly of DNA filaments called neutrophil extracellular traps (NETs), for killing pathogens. Recent data have shown that NETs actively take part in thrombus formation by interacting with RBCs, platelets and platelet adhesion molecules such as fibronectin, fibrinogen and vWF, aiding formation of the thrombus scaffold with fibrin meshwork.^21^ It has been demonstrated that older thrombi have more NETs compared with fresh thrombi.^22^

Although rare, occasionally AIS thrombi have components found in atherosclerotic plaques such as calcification, cholesterol crystals and arterial wall components.^7 10 19^ Immunostaining with CD34 has been used to identify endothelial cells in thrombi.^18 23^ Banded collagen fibres have been observed, typically at the periphery of thrombi retrieved during EVT in AIS patients, possibly an outcome of scraping the vascular wall.^9 23^ A very small proportion (1%–6%) of retrieved AIS thrombi are composed of calcified atheromatous gruel.^24^ Other clot types, such as septic emboli or emboli secondary to cardiac tumours have also been described in studies of AIS thrombi.^25^ Septic thrombi are usually characterised based on diagnosis of endocarditis or histopathological assessment of thrombus to confirm presence of pathogen.^25^

Other methods such as scanning electron microscopy (SEM), atomic force microscopy, fourier transform infrared spectroscopy (FTIR) and Raman spectroscopy have also recently been used to study AIS thrombi.^26 27^ SEM allows study of sample topology at high magnification, allowing better visualisation of the fibrin architecture and cells and their location with respect to one another.^28^

### Thrombus composition and aetiology

The TOAST (Trial of Org 10 172 in Acute Stroke Treatment) classification categorises ischaemic stroke based on aetiology into five subtypes: (1) large-artery atherosclerosis (LAA), (2) cardioembolism (CE), (3) small-vessel occlusion, (4) stroke of other determined aetiology and (5) stroke of undetermined aetiology/cryptogenic.^29^ Determining aetiology in AIS is vital since aetiology plays an important role in selection of secondary stroke prevention approaches. However, AIS patients can exhibit risk factors corresponding to both large artery atherosclerotic and cardioembolic aetiologies and the exact aetiology remains uncertain in up to 35% of AIS cases.^30^ In cryptogenic stroke, as secondary prevention treatment strategy is less clear, there may be a higher chance of recurrence. Through studying the composition of extracted thrombi, researchers are searching for novel biomarkers that could be indicative of aetiology (table 1).

The composition and structural arrangement of a thrombus, is governed by the local haemodynamic conditions during clot formation.^6 7 15^ It has been hypothesised that thrombi of arterial origin, formed on ruptured plaques in high shear stress were may be platelet rich and thrombi of cardiac origin, formed in static blood flow may be fibrin and RBC rich, but conflicting evidence to-date suggests it may not be that clear cut for cerebral thrombi.^20^ Cardioembolic and LAA clots have variously reported to have more, less, or similar levels of main components in comparison with each other (table 1).^31^ However, subsequent studies with larger datasets have observed that cardioembolic thrombi have higher fibrin content, lower RBC content and arteriogenic thrombi have higher RBC content.^8 15 32–34^

Several studies have suggested that cryptogenic strokes are primarily cardiogenic in origin, based on histological analysis^15 32^ and similar interventional and clinical outcome parameters.^8^ If on further investigation, there is clear evidence of association between thrombus composition and aetiology, this in turn could be helpful in guiding secondary stroke prevention strategies.

It has been also observed that extent of WBCs in AIS thrombi can vary^6 7 35^ and it has been suggested that WBC composition varies with aetiology. Previous studies carried out on thrombi from acute coronary syndrome highlighted the involvement of leukocytes in thrombus growth. Higher WBC percentage in cardioembolic and cryptogenic stroke clots^9^ has been reported but there have been conflicting findings reported regarding specific WBC subtypes and correlation to aetiology (table 1).^36^ Analysis of mRNA expression of inflammatory mediators found that thrombi from LAA stroke patients had significantly higher expression of IL‐1β than cardioembolic and cryptogenic thrombi.^37^ Further studies with more thrombi are needed for better understanding of role of WBCs and inflammatory mediators in AIS stroke aetiologies.

### Thrombus composition, clinical and revascularisation outcome

#### Thrombolysis

Thrombus composition can influence the efficacy of thrombolysis by r-tPA. Previous research has shown that RBC-rich thrombi respond better to r-tPA than platelet-rich or white thrombi.^38 39^

A recent study that investigated arrangement of thrombus components speculated that RBC-rich areas which have thin fibrin arrangements might be most prone to degradation by r-tPA.^5^ Platelet-rich areas within AIS thrombi had denser fibrin, and also contained a significant amount of vWF and extracellular DNA, which might play a role in r-tPA resistance of platelet-rich clots.^5^ It has been suggested that higher NETs content is associated with reperfusion resistance which might be due to formation of scaffold like structure by NETs.^40^ The potential of deoxyribonuclease 1 (DNAse 1) as a thrombolytic therapy is being explored, perhaps in combination with r-tPA.^22 40^

SEM has advanced understanding of the characteristics of thrombolysis-resistant clot which was shown to have a thick, compact outer shell made of densely compacted thrombus components including fibrin, vWF and aggregated platelets and this made the thrombi less susceptible to thrombolysis.^26^ Further study using SEM and transmission electron microscopy showed that thrombolysis-resistant thrombus contained compressed polyhedral RBCs and had a denser fibrin arrangement on the surface than non-thrombolysis treated thrombus.^28^

#### Endovascular treatment

Thrombus composition may play an important role in successful removal of thrombus via EVT. A study by Ahn *et al* did not find any correlation of thrombus components with recanalisation after EVT^15^ and a systematic review in 2016 found no association between the histopathological characteristics of thrombi retrieved during EVT and angiographic outcomes.^41^ However, recent studies have shown positive correlation between RBC content of AIS thrombi and EVT outcome (table 2).^10 33^ RBC-rich thrombi have been reported as easier to retrieve via EVT and correlate with better reperfusion rates compared with more complex fibrin/platelet-rich thrombi.^33 35^ Patients with RBC-rich thrombi had a smaller number of recanalisation manoeuvres, shorter procedure times, a shorter time interval to recanalisation.^23 33^ Fibrin-rich thrombi with low RBC content have been associated with longer EVT procedure times.^42^ Furthermore, low RBC content may be associated with secondary embolism during mechanical thrombectomy.^42^ Analysis of the composition of thrombus retrieved during each pass of an EVT device showed that erythrocyte-rich thrombus is easily retrieved whereas fibrin-rich thrombus is more resistant to retrieval.^43^

**Table 2:** Thrombus composition and clinical/revascularisation outcomes

Ref. (year)	No of patients	Staining technique	Main findings
Singh *et al* 18 2013	48	H&E, Prussian-blue, Elastica-van-Gieson, Kossa, and Periodic acid-Schiff reaction IHC:CD34 (endothelial cells)	Thrombus histology does not predict success of mechanical thrombectomy
Hashimoto *et al* 10 2016	83	H&E, Masson’s trichrome	Thrombi containing atheromatous gruel were associated with failed reperfusion Successful reperfusion associated with higher proportion of RBCs
Schuhmann *et al* 12 2016	37	H&E, MSB IHC: CD4 (T cells), CD68 (monocytes) and vWF	No association between histological findings and clinical outcome (NIHSS score) at discharge
Sporns *et al* 42 2017	180	H&E, Elastica van Gieson, Prussian blue	Fibrin rich thrombi with low RBC significantly associated with longer intervention times Thrombi with lower RBC % showed higher chances of embolisms in the thrombectomy process, suggesting a higher fragility
Funatsu *et al* 17 2019	101 (150 thrombi)	H&E, Masson’s trichrome, Elastica van Gieson staining to confirm vascular wall components	Lower RBC content, and high number of device passages associated with vascular wall component positive thrombi Successful recanalisation associated with vascular wall component negative thrombi
Douglas *et al* 16 2020	63 (91 thrombi)	MSB IHC: CD42b, vWF	Thrombus composition was not associated with stroke severity (NIHSS score ≥16) Platelet and vWF levels correlated with each other and both were inversely correlated with RBC composition Patients with platelet-rich thrombi have poorer revascularisation outcomes

IFC, immunofluorescence staining; IHC, immunohistochemical staining; MSB, Martius scarlet blue; NIHSS, National Institutes of Health Stroke Scale; RBC, red blood cell; vWF, von Willebrand factor.

The mechanical characteristics of thrombi are related to composition. An in vitro study with clots prepared from human blood found that thrombi with RBC content of 20% or above have increased viscosity and elasticity compared with clots with low RBC content.^44^ The physical characteristics of thrombi have been shown to influence the interaction of stent retrievers and aspiration catheter with thrombus.^4 41^ A study using an in vitro flow model demonstrated that a direct aspiration first-pass technique (ADAPT) with an intermediate catheter was particularly useful for RBC-rich thrombi whereas balloon guided catheter was more useful for fibrin-rich thrombi.^45^ The different frictional characteristics of thrombus components might account for the difference in response of fibrin-rich vs RBC-rich thrombi during thrombus retrieval. A study using sheep’s blood, found that fibrin rich thrombi had significantly higher coefficient of friction than RBC rich thrombi.^46^ Fibrin-rich thrombi have lesser clot integration into the thrombectomy device, making them more resistant to EVT.^47^ Dense fibrin strands have also been known to change the coefficient of friction as well as the level of physical compression.^4 46^ Furthermore, a recent study on thrombi retrieved from 19 AIS patients during EVT, showed that increased thrombus stiffness is associated with higher fibrin/platelet content.^48^ Thrombus removal is also dependent on thrombus-vessel interaction. Aged and more organised thrombi may have higher adherence to the vessel wall making removal or dissolution of the thrombus challenging.^9^ As mature, fibrin-rich thrombus is tough, sticky, and less deformable, it can be more difficult to remove with both stent retrievers and aspiration.^42 49^ Thrombus penetration is required by stent retrievers for adequate grip and this is also dependent on thrombus composition.^50^

Higher WBC percentage in thrombi has been shown to negatively correlate with recanalisation and clinical outcome such as National Institutes of Health Stroke Scale (NIHSS) score at discharge and modified Rankin Scale score upto 90 days.^9^ Studies have also shown higher NETs content in large vessel occlusions with secondary embolism and lower rates of complete recanalisation.^51^ DNA can modify the fibrin structure to make it more resistant to mechanical forces.^5^ A positive correlation between NETs percentage and device passes needed for successsful recanalisation has been reported^12^ and the presence of vWF in retrieved thrombi has also been linked to stroke severity, as measured by NIHSS scores at admission.^12 16^

Calcified emboli are quite rare in AIS patients with large vessel occlusions, but when present, calcification is associated with poor recanalisation and higher mortality.^52^ The presence of atheromatous gruel also showed a negative correlation with successful EVT recanalisation.^10^ Funatsu *et al* found a trend suggesting the presence of vascular wall components was associated with lesser recanalisation success.^17^ The occurrence of vascular wall components might be dependent on factors such as pathology of the thrombus, number of device passes and arterial location of the thrombus.^17^

Patients have the best outcomes after EVT if the entire thrombus is retrieved in a single pass.^53^ Multiple passes increase the chance of secondary embolism and poor outcome. Further research into the composition of easily fragmented thrombi and difficult to remove thrombi is needed. Advances in our understanding of the impact of thrombus composition on recanalisation outcomes will lead to new and improved EVT device development and could inform device selection in the acute clinical setting for better patient outcome.

#### Neuroimaging and thrombus composition

AIS thrombus can be identified by CT and MRI. However, studies investigating the association of thrombus imaging with recanalisation have largely utilised CT imaging (table 3). CT is more widely available than MRI. Hyperdense clots are usually RBC rich^23^ and a hyperdense artery sign (HAS) is significantly associated with RBC rich thrombi.^23 54^

**Table 3:** Studies correlating thrombus composition, clinical outcome and imaging characteristics

Ref. (year)	No of patients	r-tPA administered to eligible patients	Staining technique	Imaging technique	Main findings Revascularisation/clinical outcome imaging characteristics
Liebeskind *et al* 6 2011	50	Yes, IV r-tPA: 7 patients (14%), IA-r-tPA: 1 patient (2%)	H&E	HMCAS on NCCT and blooming artefact (BA) on GRE-MRI	RBC content determines appearance of HMCAS and BA and the absence of these signs may indicate fibrin-rich thrombi
Niesten *et al* 19 2014	22	Yes, IV r-tPA: 17 patients (77%) IA-r-tPA: 3 patients (14%)	H&E, Mallory’s phosphotungstic acid-haematoxylin (fibrin) IHC: glycophorin A (RBCs) and CD31 (platelets)	Thrombus attenuation on NCCT	Moderately positive correlation between RBC content and thrombus attenuation on NCCT
Boeckh-Behrens *et al* 9 2014	34	–	H&E, Elastica van Gieson staining	HAS on NCCT	Higher percentage of WBCs in the thrombus are associated with less favourable recanalisation(TICI<3) and clinical outcome (NIHSS score at discharge and mRS scores upto 90 days)
Kim *et al* 35 2015	37	Yes, IV r-tPA: 23 patients (62%)	H&E, IHC: glycoprotein IIIa(platelets), CD61	SVS on GRE-MRI	Higher RBC content correlates with positive SVS and negative SVS correlated with higher fibrin and platelet content
Ahn *et al* 15 2016	36	Yes, IV r-tPA: 20 patients (56%)	H&E, MSB IHC: CD42b (platelet glycoprotein Ib)	HAS on NCCT	No association between thrombus components and IV r-tPA use, HAS on NCCT No association between thrombus components and recanalisation grade
Berndt *et al* 57 2018	133		H&E	NCCT and CTA for clot perviousness	Pervious thrombi are fibrin/platelet rich
Maekawa *et al* 33 2018	43	Yes, IV r-tPA: 20 patients	H&E	Thrombus attenuation on NCCT	RBC rich thrombi are associated with higher thrombus density, and reduced procedure time
Shin *et al* 31 2018	37	Yes, IV r-tPA: 16 patients (43%)	H&E	HAS on NCCT/ blooming artefact on GRE-MRI	RBC-rich clots associated with successful recanalisation (TICI=2b/3) RBC-rich clots associated with presence of HAS/BA
Choi *et al* 39 2018	52	Yes, IV r-tPA: 52 patients (100%)	H&E, MSB	SVS on MRI	Higher % of RBCs associated with presence of SVS and better responsiveness to intravenous thrombolysis Thrombolysis responsiveness not directly associated with good clinical outcome (mRS score=0–2, after 3 months)
Fitzgerald *et al* 11 2019	85	Yes, IV r-tPA: 43 patients (51%)	MSB IFC: CD42b (platelets), fibrinogen	HAS on NCCT	Isodense clots on NCCT correlate with a high fibrin/platelet content
Fitzgerald *et al* 54 2019	50	Yes, IV r-tPA: 23 patients (46%)	H&E	HAS on NCCT	Positive correlation between RBC rich thrombi and presence of HAS
Horie *et al* 67 2019	65	Yes, IV r-tPA: 22 patients (34%)	H&E	MRI (SVS)	Stent retrievers might crush the thrombus, which may have a synergistic effect with r-tPA Thrombus histology might be altered during removal via thrombectomy No correlation between SVS and % of RBCs
Benson *et al* 58 2020	57	Yes, IV r-tPA: all patients (100%)	H&E, MSB	NCCT and CTA for clot perviousness	Pervious clots are RBC-rich whereas impervious clots are more likely to be fibrin and WBC rich

CTA, CT angiography; HAS, hyperdense arterial sign; HMCAS, hyperdense middle cerebral artery sign; IA, intra-arterial; IFC, immunofluorescence stainin; IHC, immunohistochemical staining; IV, intravenous; mRS, modified Rankin Scale; MSB, Martius scarlet blue; NCCT, non-contrast CT; NIHSS, National Institutes of Health Stroke Scale; RBC, red blood cell; r-tPA, recombinant tissue plasminogen activator; SVS, susceptibility vessel sign; TICI, Thrombolysis in Cerebral Infarction score; vWF, von Willebrand factor; WBC, white blood cell.

Isodense clots on non-contrast CT (NCCT) correlate with a high fibrin/platelet content and are more resistant to thrombolysis and EVT.^11 38^ Both NCCT and MRI can be used to recognise RBC-rich thrombi by the presence of a HAS or susceptibility vessel sign (SVS).^6 35 55^ Moreover, the density in Hounsfield units on CT images, corresponds to the thrombus composition to some degree.^23^ Clot analogues made in vitro are not able to fully represent the complexity and intra-clot variability observed in human AIS thrombi, but they have been used to assess correlation of clot composition with imaging data. RBCs have been identified as the primary variable that alter attenuation on NCCT.^55^

The vessel sign observed on gradient eco imaging (GRE) in AIS patients is called SVS. The SVS on GRE imaging is defined as a hypointense signal that spreads outside the actual thrombus periphery. The SVS is observed in 50%–85% of AIS patients with large vessel occlusion, particularly in RBC rich thrombus, while a lack of SVS usually suggests presence of fibrin-rich thrombus (table 3).^35^

Thrombus permeability or perviousness is the degree to which blood is able to flow through a thrombus structure. Thrombus perviousness is the residual flow that is quantified using simultaneous measurement of thrombus attenuation on NCCT and single-phase CT angiography, called ‘thrombus attenuation increase’ (TAI). Increased perviousness gives high TAI.^56^ Very few studies have correlated the degree of TAI with the histologic composition of thrombi retrieved during mechanical thrombectomy, however, positive correlation between clot density in NCCT and RBC content has been observed^57^ and pervious thrombi have been shown to be RBC rich, whereas impervious thrombi were more likely to be fibrin and WBC rich.^58^ Thrombus permeability might be useful as a new imaging marker for characterising thrombus and categorising stroke pathogenesis.

Imaging characteristics such as HAS and SVS have been linked to stroke aetiology. Some single centre studies have correlated SVS with cardioembolic aetiology in AIS.^35 41^ However, overall, the association of SVS with stroke aetiology has not been strongly proven. A conflicting study found no significant difference in the sensitivity of SVS for cardioembolic and LAA stroke aetiology^59^ whereas a recent large multicentre study found a significant association between SVS and cardioembolic aetiology, predicting cardioembolic aetiology with high sensitivity, but low specificity.^60^ Further studies are needed to confirm the association between SVS and cardioembolic aetiology. Although, the hyperdense middle cerebral artery sign (HMCAS) is not utilised for making therapeutic decisions, it has probable prognostic value. Prior studies have shown that HAS on NCCT correlates with better recanalisation and response to IV thrombolysis.^38^ Thrombi with HAS is likely to be RBC-rich, presenting improved recanalisation rates for both IV r-tPA and intra-arterial (IA) r-tPA.^41^ CT-based higher thrombus density has been found to be the independent predictor for secondary embolism following mechanical thrombectomy.^61^ Measuring thrombus density on NCCT could be a valuable discriminator in selecting the most appropriate reperfusion strategy for an individual patient.^62^ Additional studies are required to further investigate its role in the optimisation of thrombectomy strategy.

The prognostic value of SVS for recanalisation is debatable. SVS has been variously shown to be a negative predictor of early recanalisation after IV r-tPA treatment^63^ and more recently has also been associated with successful recanalisation.^64^ Other studies have shown that SVS is not related to success of recanalisation following intravenous r-tPA treatment or mechanical thrombectomy.^65^ However, the SVS thrombus length was demonstrated to be inversely related to success of recanalisation with stent retrievers.^65^

Thrombus permeability is potentially an important predictor of AIS treatment. It has been associated with better functional outcome, smaller final infarct volume, and higher recanalisation following IA r-tPA or IV r-tPA.^56^ A recent study analysing 408 patients from the MR CLEAN Registry, found that thrombus perviousness was associated with better functional outcome with EVT.^66^

Imaging techniques are the best way of visualising thrombus in situ, and the only way to visualise thrombi in in vivo environments. In the case of thrombi dissolved by r-tPA or thrombi not retrieved via EVT, clot imaging is the only resource for characterisation. While post thrombectomy analysis of clot composition is very valuable to improving our understanding, EVT can damage or cause structural changes in the thrombus during the removal procedure.^67^ Also, the ex vivo analysis can further alter thrombus properties via moisture loss or structural changes if a fixative is used. Despite ongoing developments and progress in AIS imaging techniques, it is not yet possible to conclude definitively regarding thrombus characteristics that could advise on the probable efficacy of thrombolysis or thrombectomy in advance of treatment. The potential to infer histological information from thrombus imaging data could be extremely valuable to clinicians in the acute stroke care setting.^55^

### New emerging tools: bioimpedance

Intraprocedural devices with dignostic capabilities or new clinical imaging approaches are needed for better guidance of device selection prior to EVT.^13^ A technique which measures characteristics of the clot prior to or during the thrombectomy procedure would give us better insights into characteristics of the clot in situ. Ideally, if thrombus composition is identified before intervention, better recanalisation with the most suitable procedural approach and device could be attained. One characteristic of tissue that can be measured via a probe is bioimpedance. By evaluating bioimpedance, thrombus characteristics could be assessed in situ, in real time prior to or during the EVT procedure.

#### Bioimpedance: basics

Biological tissues possess electrical properties and these properties are dependent on morphological, physiological and pathological conditions of the tissue and the frequency of the applied electrical signal.^68^ The cells and tissues possess endogenous (ie, active) electrical properties (due to ionic activities inside cells) as well as passive electrical properties (due to stimulation via external electrical signal). One such property, is the ability of cells or the biological tissue to oppose the applied current, called bioimpedance.^68^ To measure bioimpedance, an excitation signal is applied between two electrodes in the form of current or potential and the resultant response is measured using same or different electrodes.^69^ Electrical impedance (Z), defined as the ratio between voltage (V) and current (I), applies to the alternating current (AC), and the resistive, capacitance or inductive components of the tissue all contribute to the measured impedance.

The bioimpedance measured with applied alternating current varies with the frequency^69^ and depending on the range used, (low frequency, radiofrequency or microwave) the impedance can give information on the physiological, morphological and pathological conditions of the tissue.^70^ Indeed, the cell membranes of the tissue can be considered as a capacitor, and as such, they can act as an insulator at low frequencies (generally below 40 KHz). The current passes between the cells revealing information about the structure of the tissue. At a higher frequency (generally around 1000 KHz), the current passes through the cells, revealing information about the cells themselves.

#### Electrochemical impedance spectroscopy for thrombus characterisation

Electrical Impedance Spectroscopy (EIS) is carried out by measuring the electrical impedance of biological tissues over a frequency range. Since the electrical response of tissues is determined by their cellular components and the dimension, internal structure and arrangements of the constituent cells, tissues with different morphological and physiological properties give rise to characteristic impedance spectra.^70^ EIS has been used in a variety of applications such as monitoring of cell cultures, characterisation of biological cells and tissue engineering applications.^70^

Based on EIS principles, clinical applications have been developed, especially in the field of oncology. SciBase, Dilon Technologies, Zilico, are some of the companies that developed medical devices with CE and/or FDA (US Food and Drug Administration) approval. These devices (Nevisense, MarginProbe, ZedScan) can discriminate healthy tissues from cancerous tissues (respectively, skin, breast and cervical) with high sensitivity and specificity.^71–73^ The EIS-based devices use multiple sensors which are in direct contact with the tissue to be probed, avoiding electrical noise coming from other biological components interposed between the electrodes and the tissue itself. This enhances the capability of detecting the electrical properties of the tissues that reflect its morphological and pathological structure. In addition, the EIS measures the impedance between two nearby electrodes using a wide range of frequency. Since the size of electrodes can be as small as few hundred micrometres, this allows precise local measurements to discriminate between normal and pathological tissue.

EIS-based sensors are being developed for blood components such as WBCs^74^ and NETs.^75^ Studies have also shown that aggregation of RBCs^76^ influences EIS measurements. Few studies have used EIS for thrombus detection.^77 78^ One study estimated the risk of thrombus formation by detecting circulating platelet derived microparticles^78^ whereas another study utilised dielectric relaxation method to detect thrombosis in real time using bovine blood.^77^ A recent study by Santorelli *et al* demonstrated that based on EIS measurements, blood clot analogues can be classified into RBC rich or platelet and fibrin rich clots.^79^ This promising observation suggests EIS may be useful to detect AIS thrombus characteristics in the acute care setting, although considerable further research is needed to translate this observation to real AIS thrombi in situ and in thrombi of heterogeneous composition.

Some studies have also demonstrated the feasibility of utilising balloon catheters mounted with microelectrodes for EIS measurements. The integration of four microelectrodes onto a catheter is made possible using a flexible and ultralight polyimide foil. Using such a system in atherosclerotic animal models, intravascular EIS measurements differed significantly in aortic plaques compared with normal aortic tissues.^80^ More recently, an EIS sensor with six microelectrodes cirncumferentially mounted on an inflated balloon catheter to optimise contact with the endothelium wall surface was successfully used for 3D mapping and detection of atherosclerotic lesions in a rabbit model.^81^ These studies emphasise the possibility of development of a miniaturised EIS based sensor integrated to a medical device such as catheter or guidewire. Such a device could be used to characterise thrombi in AIS patients, potentially reducing intervention time and improving outcome.

## Conclusion

In recent years, mechanical thrombectomy has facilitated the analysis of thrombi retrieved during the EVT procedure and marked heterogeneity has been observed. Restricted availability of thrombi other than ones found in LVOs that are easily retrievable limits the current knowledge of AIS thrombi. Further studies will improve understanding of aetiology and its correlation to thrombus composition and clinical outcome. New emerging methods such as EIS, could be beneficial for gaining new insights into pathophysiological mechanisms of thrombus formation and identifying clot characteristics in situ in the acute care setting, aiding in selection of better treatment options for the AIS patients.
